# Iron Supplementation and Mortality in Incident Dialysis Patients: An Observational Study

**DOI:** 10.1371/journal.pone.0114144

**Published:** 2014-12-02

**Authors:** Emanuel Zitt, Gisela Sturm, Florian Kronenberg, Ulrich Neyer, Florian Knoll, Karl Lhotta, Günter Weiss

**Affiliations:** 1 Department of Nephrology and Dialysis, Feldkirch Academic Teaching Hospital, Feldkirch, Austria; 2 Vorarlberg Institute for Vascular Investigation and Treatment (VIVIT), Feldkirch, Austria; 3 Department of Dermatology and Venereology, Innsbruck Medical University, Innsbruck, Austria; 4 Division of Genetic Epidemiology, Department of Medical Genetics, Molecular and Clinical Pharmacology, Innsbruck Medical University, Innsbruck, Austria; 5 Department of Internal Medicine VI (Infectious Diseases, Immunology, Rheumatology, Pneumology), Innsbruck Medical University, Innsbruck, Austria; Lady Davis Institute for Medical Research/McGill University, Canada

## Abstract

**Background:**

Studies on the association between iron supplementation and mortality in dialysis patients are rare and conflicting.

**Methods:**

In our observational single-center cohort study (INVOR study) we prospectively studied 235 incident dialysis patients. Time-dependent Cox proportional hazards models using all measured laboratory values for up to 7.6 years were applied to study the association between iron supplementation and all-cause mortality, cardiovascular and sepsis-related mortality. Furthermore, the time-dependent association of ferritin levels with mortality in patients with normal C-reactive protein (CRP) levels (<0.5 mg/dL) and elevated CRP levels (≧0.5 mg/dL) was evaluated by using non-linear P-splines to allow flexible modeling of the association.

**Results:**

One hundred and ninety-one (81.3%) patients received intravenous iron, 13 (5.5%) patients oral iron, whereas 31 (13.2%) patients were never supplemented with iron throughout the observation period. Eighty-two (35%) patients died during a median follow-up of 34 months, 38 patients due to cardiovascular events and 21 patients from sepsis. Baseline CRP levels were not different between patients with and without iron supplementation. However, baseline serum ferritin levels were lower in patients receiving iron during follow up (median 93 *vs* 251 ng/mL, p<0.001). Iron supplementation was associated with a significantly reduced all-cause mortality [HR (95%CI): 0.22 (0.08–0.58); p = 0.002] and a reduced cardiovascular and sepsis-related mortality [HR (95%CI): 0.31 (0.09–1.04); p = 0.06]. Increasing ferritin concentrations in patients with normal CRP were associated with a decreasing mortality, whereas in patients with elevated CRP values ferritin levels>800 ng/mL were linked with increased mortality.

**Conclusions:**

Iron supplementation is associated with reduced all-cause mortality in incident dialysis patients. While serum ferritin levels up to 800 ng/mL appear to be safe, higher ferritin levels are associated with increased mortality in the setting of concomitant inflammation.

## Introduction

Imbalances of iron homeostasis are a frequent finding in dialysis patients. These are due to true iron deficiency caused by gastrointestinal bleeding [1], blood loss with every hemodialysis session [2], decreased duodenal iron absorption [3] and by iron demand from the use of erythropoiesis-stimulating agents (ESA) [4]. Chronic inflammation of multiple causes in these patients results in impaired duodenal iron absorption and stimulates macrophage iron retention through the combined activities of the acute-phase protein hepcidin and cytokines [5]. All these mechanisms lead to iron-restricted erythropoiesis, which aggravates anemia due to the lack of erythropoietin found in association with chronic kidney disease. Intravenous iron supplementation and ESA have been used to increase hemoglobin levels in dialysis patients, decrease the need for red blood cell transfusions and improve patients' general quality of life [6]–[8]. Specifically, iron supplementation is necessary to correct true iron deficiency, prevent its development in ESA-treated patients [9], increase responsiveness to ESA, and reduce ESA dosages [10].

Studies investigating the effect of iron supplementation on mortality of dialysis patients have produced conflicting results. Whereas two studies found a higher mortality rate in patients treated with high iron dosages [11], [12], another study could not confirm these observations [13]. No prospective randomized controlled trials of iron supplementation evaluating solid clinical end-points are available so far. Iron supplementation has been linked to infection, increased oxidative stress and atherosclerosis, thereby limiting its uncritical use [14]–[18]. Unfortunately, long-term safety data on the effect of iron supplementation on such important clinical endpoints are lacking.

This study aimed to investigate the association between iron supplementation and all-cause mortality, cardiovascular and sepsis-related mortality in a well-characterized inception cohort of incident dialysis patients who were followed for up to more than seven years.

## Subjects and Methods

### Patient population

The INVOR Study [19], [20] (Study of Incident Dialysis Patients in Vorarlberg) is a single-centre, prospective, observational cohort study of incident dialysis patients in Vorarlberg, Austria's westernmost state with approximately 400,000 inhabitants. All incident dialysis patients starting chronic dialysis treatment between May 1, 2000 and April 30, 2006 were enrolled. Patients with a malignant tumor at initiation of dialysis were excluded. A total of 235 patients were included and followed up for a maximum of 7.6 years until December 31, 2007 or until death. Four patients were lost to follow-up, three of whom regained renal function and one who moved away. Type of and change in renal replacement therapy (HD or PD, transplantation) were recorded and considered as time-dependent treatment status for data analysis. Patients were treated according to the European Best Practice Guidelines in place at the time of treatment. Ferric gluconate was routinely used as intravenous iron supplement. Intravenous iron (ferric gluconate) was given as continuous maintenance therapy generally once per week in varying doses (range: 12.5 mg (minimum dose) to 62.5 mg (maximum dose). With increasing ferritin concentrations (>500 ng/mL) physicians usually halted the iron application and re-started it again after a significant ferritin drop. In peritoneal dialysis patients or kidney transplant recipients either ferrous sulfate or ferric gluconate was used as oral iron supplementation. ESAs used included epoetin alfa, epoetin beta or darbepoetin.

The study was approved by the ethic committee of the Innsbruck Medical University and by the ethical review committee of the State of Vorarlberg. All patients enrolled in the study provided written informed consent.

### Data description

As described recently [19], [20] clinical, laboratory and medication data were collected prospectively, starting at initiation of dialysis. All-cause mortality data including causes of death (autopsy-proven in 33%) were evaluated. Laboratory parameters were measured in a central laboratory and continuously recorded during the study period. Full blood count, phosphorus, creatinine and calcium were measured monthly, and albumin, C-reactive protein (CRP), ferritin, and iPTH every three months. All available measurements of ferritin, CRP, albumin and hemoglobin were used in the time-dependent Cox regression modeling described below.

### Study outcomes

The outcomes of interest were all-cause mortality as well as cardiovascular or sepsis-related mortality.

### Statistical Methods

At baseline, categorical data were compared using the χ^2^ test, and continuous variables were analyzed using an unpaired T-test or the nonparametric Mann-Whitney U test. Data are presented as mean±SD and as median and 25^th^ and 75^th^ percentiles for skewed variables, where appropriate.

To investigate the influence of iron supplementation on mortality, a time-dependent Cox proportional hazards model was used allowing all variables to vary over different measurements during the whole observation period for each patient. Thus, each time interval between two successive measurements enters the model independently. Each covariate that entered the model was updated at the time it was measured and modeled in a time-dependent fashion. If not all variables were measured at a particular date, the value measured at the last observation of the variable was used in its place (“last observation carried forward”). The proportional hazards assumption was analyzed for each model by testing for zero slopes of scaled Schoenfeld residuals. The Cox models were adjusted for age, sex, diabetes mellitus and the time-dependent variables type of renal replacement therapy, CRP, albumin and hemoglobin. An additional analysis was conducted in patients with and without diabetes mellitus with the same adjustments except for diabetes. Adjusted survival curves were plotted for iron supplementation, holding all covariates fixed at their mean level.

The influence of ferritin levels on mortality was investigated in all patients and also in subjects with CRP levels <0.5 mg/dL indicating normal CRP *versus* those ≧0.5 mg/dL indicating elevated CRP during follow-up. Additional sensitivity analyses were performed with higher CRP cut-offs (<1 or ≧1 mg/dL, <5 or ≧5 mg/dL). In order to evaluate the effects of ferritin levels on clinical outcome parameters, non-linear P-splines of degree 3 were estimated. A spline of degree 3 is a linear combination of cubic functions that can fit virtually any smooth curve to the data. Therefore, the analysis was not restricted to a potential linear relationship between ferritin and mortality. The spline term can be split into its linear and non-linear components, which can each be tested separately. For the linear term, a HR can be estimated, whereas the non-linear component can be depicted in a plot of the log(HR). Cox models were calculated univariately including the time-dependent ferritin measurements linearly with calculation of hazard ratios (HR) for a ferritin increment of 100 ng/mL and additionally adjusted for age and sex. An extended model was also used, additionally adjusting for diabetes mellitus and the time-dependent serum concentrations of albumin and hemoglobin.

All analyses were conducted with the IBM SPSS Statistics version 20 (IBM Corp., NY, USA) and R using the “survival” package.

## Results

### Patient baseline characteristics

Table 1 presents the baseline demographic and laboratory characteristics as well as medication and comorbidities of the 235 incident dialysis patients at the start of dialysis treatment and during follow-up. Median follow-up time was 2.8 years. Of the entire cohort 204 (86.8%) patients received iron supplementation throughout the observation period. Intravenous ferric gluconate was administered at a mean monthly dose of 209 mg in 191 (81.3%) patients during a mean observation period of 44.6±22.6 months. During a mean follow-up of 37.5±21.6 months 13 (5.5%) patients received ferrous sulfate or ferric gluconate orally at a mean monthly dose of 1455 mg. During follow-up 58 patients (24.7%) received a kidney transplant, 49 of them had previously received intravenous iron therapy during hemodialysis. Mean (±SD) duration between initiation of dialysis and kidney transplantation in these patients was 33±18.6 months. Most of the baseline laboratory parameters did not differ between patients with and without iron supplementation during follow-up. Compared to the group of patients with iron supplementation, the group without iron therapy included a greater proportion of peritoneal dialysis patients (48.4 *vs* 11.3%, p<0.001) and fewer patients with diabetes (16.1 *vs* 37.7%, p<0.05). At initiation of dialysis 191 (81.3%) patients already received ESA. During follow-up, patients with iron supplementation received higher weekly ESA doses compared to patients without iron supplementation (median (25^th^, 75^th^ percentile): 7349 (4660, 10562) epoetin units/week *vs* 4008 (1192, 8047) epoetin units/week, p = 0.01). Baseline CRP levels did not differ between patients with and without iron supplementation (median 0.89 *vs* 1.52 mg/dL, p = 0.1), whereas baseline serum ferritin levels (median 93 *vs* 251 ng/mL, p<0.001) and transferrin saturation (15% *vs* 22%, p<0.01) were lower in patients receiving iron during follow up (Table 1).

**Table 1:** Clinical characteristics of patients at baseline and during follow-up stratified for iron supplementation during follow-up.

	All patients	Iron supplementation	No iron supplementation
	(n = 235)	(n = 204)	(n = 31)
Sex			
Male, n (%)	146 (62.1%)	126 (61.8%)	20 (64.5%)
Female, n (%)	89 (37.9%)	78 (38.2%)	11 (35.5%)
Age (years)	61.7±14.0	61.7±13.7	62.0±16.0
Body Mass Index (kg/m^2^)	26.1±4.5	26.2±4.6	25.2±3.8
Start of dialysis with			
Hemodialysis, n (%)	197 (83.8%)	181 (88.7%)	16 (51.6%) d
Peritoneal dialysis, n (%)	38 (16.2%)	23 (11.3%)	15 (48.4%) d
Year of start of dialysis			
2000–2003	122 (51.9%)	106 (52.0%)	16 (51.6%)
2004–2006	113 (48.1%)	98 (48.0%)	15 (48.4%)
Diabetes mellitus, n (%)	82 (34.9%)	77 (37.7%)	5 (16.1%) a
Systolic blood pressure (mmHg)	154.0±22.7	153.9±22.6	154.6±23.5
Diastolic blood pressure (mmHg)	83.0±12.3	82.7±12.6	82.9±11.9
	[78.0; 84.0; 90.0]	[77.0; 83.0; 90.0]	[78.0; 85.0; 91.0]
***Medication at baseline***			
ESA, n (%)	183 (77.9%)	164 (80.4%)	19 (61.3%) a
Iron supplements, n (%)	53 (22.6%)	53 (26.0%)	0 (0.0%) c
ESA and iron supplements, n (%)	51 (21.7%)	51 (25.0%)	0 (0.0%) c
***Laboratory parameters at baseline***			
Ferritin (ng/mL)	174±207	155±192	320±251 d
	[44; 111; 234]	[42; 93; 195]	[188; 251; 416]
Iron (µg/dl)	51.4±31.5	50.0±30.8	62.4±34.8 a
	[30.0; 44.0; 61.0]	[30.0; 41.0; 61.0]	[42.8; 53.5; 79.0]
Transferrin (mg/dL)	202.5±44.5	204.4±44.0	188.3±46.5
Transferrin saturation (%)	18.6±11.3	17.8±10.7	24.1±13.8 b
	[10.9; 16.0; 22.8]	[10.0; 15.0; 22.0]	[14.6; 21.7; 28.0]
Hemoglobin (g/dL)	11.17±1.72	11.17±1.67	11.12±1.99
C-reactive protein (mg/dL)	3.24±5.34	3.21±5.50	3.45±4.20
	[0.30; 0.98; 3.00]	[0.30; 0.89; 2.79]	[0.57; 1.52; 4.81]
Creatinine (mg/dL)	7.28±2.64	7.23±2.65	7.67±2.54
	[5.50; 6.80; 8.60]	[5.50; 6.70; 8.35]	[5.60; 8.20; 8.70]
Albumin (g/dL)	3.71±0.65	3.72±0.65	3.65±0.69
Calcium (mg/dL)	8.5±1.1	8.3±1.1	8.7±1.0
Phosphorus (mg/dL)	6.1±1.9	6.0±1.9	5.6±1.9
iPTH (pg/mL)	350.5±264.9	360.5±273.6	279.9±180.2
	[156.5; 287.2; 468.7]	[159.4; 292.7; 468.7]	[147.9; 222.9; 397.0]
Bicarbonate (mEq/L)	21.00±3.59	20.93±3.44	21.75±4.98
Erythrocytes (T/L)	3.73±0.62	3.75±0.62	3.61±0.65
Leukocytes (G/L)	8.18±3.29	8.27±3.26	7.64±3.43
	[6.10; 7.40; 9.90]	[6.20; 7.50; 9.95]	[5.43; 7.05; 8.83]
Total cholesterol (mg/dL)	189.8±51.0	189.7±50.7	190.3±54.7
***Comorbidities at baseline***			
CAD *, n (%)	40 (17.0%)	36 (17.6%)	4 (12.9%)
CVD **, n (%)	70 (29.8%)	63 (30.9%)	7 (22.6%)
PAD ***, n (%)	40 (17.0%)	35 (17.2%)	5 (16.1%)
***Follow-up***			
ESA, n (%)	234 (99.6%)	204 (100.0%)	29 (93.5%)
Follow-up time (months) ‡	38.2±23.2	40.4±22.6	24.0±22.4 d
Transplantation, n (%)	58 (24.7%)	49 (24.0%)	9 (29.0%)
All-cause mortality, n (%)	82 (34.9%)	66 (32.4%)	16 (51.6%) a
CV mortality #, n (%)	38 (16.2%)	32 (15.7%)	6 (19.4%)
Sepsis mortality, n (%)	21 (8.9%)	16 (7.8%)	5 (16.1%)
CV and/or sepsis mortality, n (%)	59 (25.1%)	48 (23.5%)	11 (35.5%)

Mean ±SD [25^th^, 50^th^ and 75^th^ percentile for cases of non-normal distribution] or number (%).

a p<0.05;

b p<0.01;

c p<0.005;

d p<0.001, comparison between patients who ever received iron supplementation and patients who never received iron supplementation during the observation period.

* **Coronary artery disease (CAD)**: myocardial infarction (MI), percutaneous transluminal coronary angioplasty (PTCA), aortocoronary bypass (ACBP).

** **Cardiovascular disease (CVD)**: myocardial infarction (MI), percutaneous transluminal coronary angioplasty (PTCA), aortocoronary bypass (ACBP), coronary artery stenosis ≥50%, ischemic cerebral infarction, transient ischemic attack (TIA)/PRIND.

*** **Peripheral arterial disease (PAD)**: vascular stenosis, percutaneous transluminal angioplasty (PTA), peripheral bypass, amputation.

‡ Follow-up time was calculated as the time from the start of dialysis until the patient died or the end of the observation period was reached.

# **Cardiovascular mortality**: myocardial infarction (MI), heart failure, sudden death, ischemic stroke, hemorrhagic stroke.

### Association between iron supplementation and mortality

**Figure 1:**
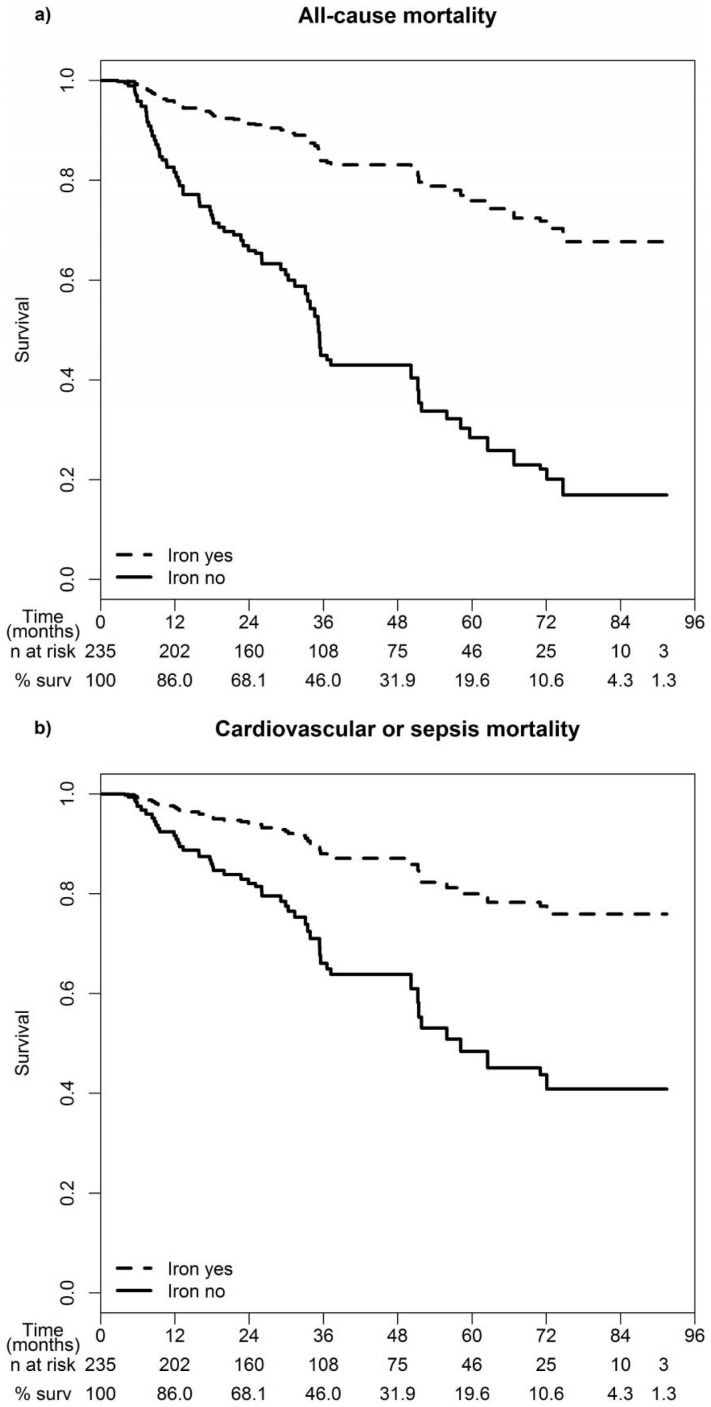
Survival curves for a) all-cause mortality and b) cardiovascular or sepsis-related mortality stratified for iron supplementation. Adjusted for age, sex, time-dependent type of renal replacement therapy, diabetes, time-dependent C-reactive protein, albumin and hemoglobin. The number of patients at risk for each year of observation is given with the last observation time at 91 months. “% surv” indicates the percentage of survivors for each 12-month interval.

During a median follow-up of 34 months 82 (34.9%) patients died, 38 from cardiovascular diseases and 21 from sepsis. Adjusted survival curves showed significantly better survival in patients receiving iron supplementation along with a lower cardiovascular and sepsis-related mortality as compared to subjects not receiving iron (Figure 1).

Table 2 presents results from time-dependent Cox proportional hazard models according to absence/presence of iron supplementation for all-cause mortality and cardiovascular or sepsis-related mortality. A significantly lower all-cause mortality was found in patients receiving iron supplementation [HR (95% CI): 0.22 (0.08–0.58); p = 0.002]. The association between iron supplementation and decreased risk of cardiovascular or sepsis-related mortality was of borderline significance [HR (95% CI): 0.31 (0.09–1.04); p = 0.06] (Table 2). We additionally evaluated the association between iron supplementation and all-cause mortality stratified for the presence of diabetes mellitus. Whereas a significant risk reduction [HR (95% CI): 0.19 (0.06–0.56); p = 0.002] was found in non-diabetic patients with iron supplementation (n = 127) as compared to not iron supplemented non-diabetic patients (n = 26), this effect was not observed in diabetic patients (n = 77 with iron supplementation *vs* n = 5 without iron therapy [HR (95% CI): 0.51 (0.02–1.59); p = 0.7]). However, due to the low number of subjects in the latter group (n = 5) with only one event occurring in this subgroup the significance of this analysis is limited (Table 3).

**Table 2:** Association between iron supplementation and all-cause mortality and cardiovascular or sepsis-related mortality using time-dependent Cox proportional hazards models*.

	All-cause mortality			CV or sepsis mortality**		
	HR	(95% CI)	P-value	HR	(95% CI)	P-value
Age (years)	1.05	(1.03–1.08)	<0.001	1.04	(1.02–1.07)	<0.001
Sex						
Female	Ref.			Ref.		
Male	1.14	(0.68–1.90)	0.6	1.38	(0.73–2.60)	0.3
Type of renal replacement therapy						
Hemodialysis	Ref.			Ref.		
Peritoneal dialysis	0.28	(0.07–1.03)	0.06	0.55	(0.14–2.12)	0.4
Transplantation	0.47	(0.14–1.54)	0.2	0.38	(0.08–1.72)	0.2
Diabetes mellitus						
No	Ref.			Ref.		
Yes	1.31	(0.81–2.12)	0.3	1.54	(0.87–2.73)	0.1
Iron supplementation						
No	Ref.			Ref.		
Yes	0.22	(0.08–0.58)	0.002	0.31	(0.09–1.04)	0.06
C-reactive protein (mg/dL)	1.13	(1.10–1.17)	<0.001	1.11	(1.07–1.15)	<0.001
Albumin (g/dL)	0.33	(0.21–0.50)	<0.001	0.31	(0.18–0.53)	<0.001
Hemoglobin (g/dL)	0.95	(0.81–1.12)	0.5	0.90	(0.75–1.08)	0.3

* Adjusted for age, sex, diabetes mellitus and the time-dependent variables type of renal replacement therapy, C-reactive protein, albumin and hemoglobin.

** **Cardiovascular or sepsis mortality**: myocardial infarction (MI), heart failure, sudden death, ischemic stroke, hemorrhagic stroke, sepsis.

**Table 3:** Association between iron supplementation and all-cause mortality in patients with diabetes mellitus and without diabetes mellitus using time-dependent Cox proportional hazards models*.

	All-cause mortality					
	Diabetes			Non-Diabetes		
	HR	(95% CI)	P-value	HR	(95% CI)	P-value
Age (years)	1.03	(0.99–1.07)	0.2	1.07	(1.03–1.10)	<0.001
Sex						
Female	Ref.			Ref.		
Male	0.98	(0.45–2.11)	0.9	1.08	(0.51–2.31)	0.8
Type of renal replacement therapy						
Hemodialysis	Ref.			Ref.		
Peritoneal dialysis	0.76	(0.07–8.73)	0.8	0.30	(0.08–1.15)	0.08
Transplantation	-	-	-	0.63	(0.14–2.82)	0.5
Iron supplementation						
No	Ref.			Ref.		
Yes	0.51	(0.02–1.59)	0.7	0.19	(0.06–0.56)	0.002
C-reactive protein (mg/dL)	1.09	(1.04–1.14)	0.001	1.17	(1.12–1.22)	<0.001
Albumin (g/dL)	0.24	(0.11–0.49)	<0.001	0.40	(0.22–0.71)	0.002
Hemoglobin (g/dL)	0.79	(0.63–0.97)	0.03	1.09	(0.89–1.35)	0.4

* Adjusted for age, sex and the time-dependent variables type of renal replacement therapy, C-reactive protein, albumin and hemoglobin.

### Association between ferritin and mortality as a function of time-dependent CRP levels

**Figure 2:**
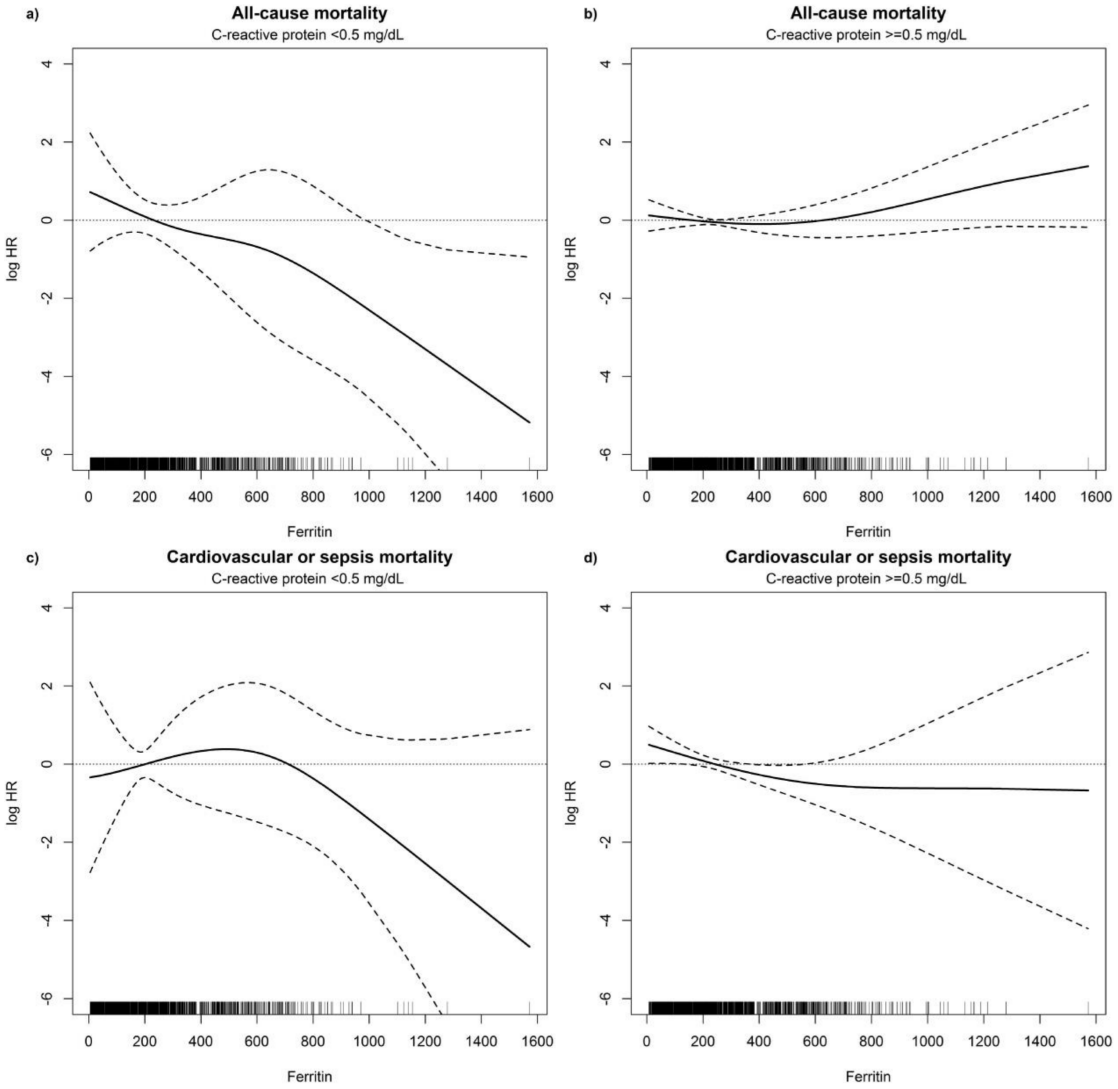
Cox regression results. P-splines to explore the functional form of the effect of ferritin values (ng/mL) on the log hazard ratio for the risk of all-cause mortality and cardiovascular or sepsis-related mortality in patients with C-reactive protein <0.5 mg/dL and ≥0.5 mg/dL during follow-up, adjusted for age, sex, diabetes mellitus and time-dependent albumin and hemoglobin. Dashed lines are the pointwise 95% CI. The rugplot at the bottom of the figures displays the number of measurements.

Adjusted non-linear P-splines demonstrated an association between time-dependent ferritin values and all-cause mortality as well as cardiovascular or sepsis-related mortality (Table 4). When studying patients with a time-dependent normal CRP level (<0.5 mg/dL) during follow-up a significant linear inverse relationship was found between increasing ferritin values and decreasing all-cause mortality (Figure 2a). In contrast, in patients with time-dependent increased CRP concentrations (≧0.5 mg/dL) during follow-up all-cause mortality increased with increasing ferritin levels (Figure 2b). The linear component of the non-linear spline was highly significant in patients with normal CRP concentrations (p<0.001). In the group of patients with elevated CRP levels the linear component was of borderline overall significance (p = 0.06), with a significantly increased risk of death in subjects with ferritin levels>800 ng/mL (HR: 2.57, p = 0.047). The non-linear component was significant in both groups (p = 0.04 and p = 0.001, respectively). Figure 2c shows a trend toward a decreasing risk for cardiovascular or sepsis-associated mortality in patients with higher ferritin values and normal CRP during follow-up, but neither the linear nor the non-linear component of the P-spline was significant. In patients with CRP levels ≧0.5 mg/dL during follow-up a significant non-linear inverse relationship (p<0.001) was observed between decreasing cardiovascular or sepsis-related mortality and ferritin values up to 600 ng/mL (Figure 2d). Sensitivity analyses with higher CRP cut-offs (<or ≧1 mg/dL, <or ≧5 mg/dL) supported our findings of a significantly increased mortality risk with increasing ferritin levels in patients with higher CRP values (Tables S1 and S2, Figures S1 and S2).

**Table 4:** Association between time-dependent ferritin and all-cause mortality and cardiovascular or sepsis-related mortality in patients with C-reactive protein <0.5 mg/dL and ≧0.5 mg/dL during follow-up using time-dependent Cox proportional hazards models.

			CRP<0.5 mg/dL						CRP≧0.5 mg/dL					
Ferritin per 100 ng/mL increase			All-cause mortality			CV or sepsis mortality**			All-cause mortality			CV or sepsis mortality**		
			(n events = 9)			(n events = 4)			(n events = 71)			(n events = 54)		
			HR	(95% CI)	P-value	HR	(95% CI)	P-value	HR	(95% CI)	P-value	HR	(95% CI)	P-value
**Non-linear effect modeling using P-splines**														
Adjustment:	None	Linear part	0.68	(0.62–0.74)	<0.001	0.77	(0.67–0.88)	<0.001	1.15	(1.08–1.23)	<0.001	1.05	(0.97–1.14)	0.2
		Non-linear part			0.07			0.05			<0.001			<0.001
	Age, sex	Linear part	0.68	(0.59–0.78)	<0.001	0.84	(0.70–1.01)	0.07	1.15	(1.06–1.25)	<0.001	1.05	(0.96–1.15)	0.3
		Non-linear part			0.05			0.05			0.001			0.001
	Extended*	Linear part	0.67	(0.59–0.77)	<0.001	0.85	(0.68–1.05)	0.1	1.08	(1.00–1.18)	0.06	0.99	(0.90–1.08)	0.8
		Non-linear part			0.04			0.06			0.001			<0.001

Shown for each model are estimated HRs for the linear component of the non-linear P-spline and HRs for ferritin measurements per 100 ng/mL increase.

* Adjusted for age, sex, diabetes mellitus and time-dependent albumin and hemoglobin.

** **Cardiovascular or sepsis mortality**: myocardial infarction (MI), heart failure, sudden death, ischemic stroke, hemorrhagic stroke, sepsis.

In patients with iron supplementation a total of 4556 CRP measurements were collected. The median CRP levels did not differ between periods with (2053 CRP measurements, 0.76 mg/dL) and without iron supplementation (2503 CRP measurements, 0.73 mg/dL, p = 0.8).

## Discussion

In our prospective long-term observational study we found a significant and independent association between iron supplementation and reduced all-cause mortality in incident dialysis patients during a follow-up period of up to seven years. Time-dependent Cox regression analyses were used including all information on adjusted laboratory parameters available from the entire observation period.

Studies investigating the effect of iron supplementation on solid clinical outcome data including survival over a long observation period are scarce. An earlier report using a retrospective database from a historical dialysis cohort found improved survival with intravenous iron at dosages of up to 400 mg/month, whereas doses >400 mg/month were associated with slightly increased mortality rates over a period of two years [12]. A recent analysis of the international Dialysis Outcomes and Practice Patterns Study (DOPPS) found an increased mortality in dialysis patients given intravenous iron doses of ≧300 mg/month over four months compared to patients receiving lower doses [21]. Another retrospective study found an increased mortality risk with cumulative iron doses of>1000 mg during a two year follow-up. After using time-dependent models adjusting for varying iron dosing and changing morbidity during the study the increased mortality risk could no longer be found, not even for cumulative iron doses of >1800 mg per six month [13]. Our data generated in a cohort of incident dialysis patients over a much longer follow-up period support these earlier findings. We found that the survival benefit in the patients with iron supplementation was independent from changes in hemoglobin levels. When adjusting for time-dependent alterations of CRP and albumin concentrations as surrogates of inflammation and malnutrition, we confirmed the independent association between lower mortality and iron supplementation. Using the entire data we gained an informative depth of data over a very long observation period. The time-dependent modelling we applied has several advantages since it does not consider only one or a few data points but the full spectrum of data in each patient. It furthermore avoids censoring of data in case of change of renal replacement status since this new phase of treatment can be considered as time-dependent covariate. Censoring of the data has major disadvantages such as loss of power due to loss of follow-up time and informative bias due to the health status influencing the choice of renal replacement therapy and probability of transplantation.

The group without iron supplementation included a significantly higher proportion of PD patients. Possible explanations for this finding could be the reduced blood loss, better residual renal function and less chronic inflammation as compared to hemodialysis patients [22]. However, we adjusted for the time-dependent type of renal replacement therapy, which was of borderline significance for all-cause mortality (Table 2 and Figure 1). As most of our patients received iron intravenously (94% of all patients receiving iron), our results mainly demonstrate the effect of intravenous iron supplementation in incident dialysis patients. The mean monthly iron dose used in our study is very well in line with the reported monthly doses in DOPPS Phase 3, indicating general practice pattern [23].

Anemia correction trials have shown a significant association between higher ESA doses and increased all-cause mortality [24], [25]. Usually, patients without iron supplementation require higher ESA doses. In our cohort, patients without iron supplementation received lower ESA doses compared to the group with iron supplementation. Therefore, the higher mortality risk in our patients without iron supplementation is not caused by the applied ESA dose.

It was recently suggested, that low dose continuous iron maintenance therapy might be preferred over high-dose bolus application due to a lower infection-related hospitalization and death risk [26]. In our study a continuous maintenance therapy was uniformly used, therefore we cannot exclude differences in all-cause and cardiovascular mortality depending on the iron dosing regimen. However, the same group found no association between higher iron doses or bolus application and short-term cardiovascular morbidity and mortality in the same cohort [27]. Ferric gluconate was applied exclusively as intravenous iron supplement over the complete observation period in our cohort. This could be regarded as an advantage excluding varying effects caused by different iron preparations. On the other hand, it also limits the applicability of our findings to ferric gluconate only. Recently, new physicochemically more stable iron preparations have been introduced into clinical practice. All intravenous iron supplements have proved to be effective compared to oral or no iron supplementation [28]. Prospective randomized head-to-head comparisons with clinically relevant end-points between these different iron preparations are missing. Recently published observational DOPPS data showed an increased mortality with monthly intravenous iron doses>300 mg irrespective of the applied iron preparation [26]. Experimental *in vitro* and *ex vivo* data found monocytic iron accumulation as a function of circulating hepcidin levels and inflammation following intravenous iron administration and in addition an impaired monocytic immune function, cytokine expression and cell differentiation and an increased formation of reactive oxygen species with the less stable iron preparations ferric gluconate and iron sucrose, which was not observed with the more stable ferric carboxymaltose, iron isomaltoside 100 and ferumoxytol [29]–[31]. Therefore, substance-specific biological side-effects and toxicity may theoretically differ *in vivo* as a function of the iron preparation and backbone used with potential effects on patients' morbidity [32]. This hypothesis could only be tested in a randomized controlled trial comparing different intravenous iron preparations [33]. Due to the paucity of prospective controlled data, such trials exploring the effect of intravenous iron supplementation on morbidity and mortality of dialysis patients and in addition by comparing different iron preparations are urgently needed to improve the scientific evidence for our clinical work. Recently, such a study has been started, called PIVOTAL, which is currently recruiting a target of 2080 patients from over 50 sites in the UK, and which is comparing the effects of a proactive high-dose, with a reactive low-dose regimen of intravenous iron sucrose in ESA-treated hemodialysis patients (https://www.clinicaltrialsregister.eu/ctr-search/trial/2013-002267-25/GB#F).

Is there a reasonable explanation for a survival benefit upon iron supplementation in dialysis patients, although iron may be harmful by impairing the immune response to microorganisms and promoting free radical production? Iron blocks the stimulatory effect of interferon-γ on monocytes, thereby reducing secretion of the pro-inflammatory cytokine TNF-α [34]. In a small study in hemodialysis patients with baseline ferritin levels of 300 ng/mL weekly application of 100 mg intravenous iron over 12 weeks indeed caused a progressive decrease in TNF-α and an increase in the anti-inflammatory cytokine interleukin-4 [35]. In addition, endogenous peroxides decreased, possibly as a consequence of reduction of TNF-α, which is a potent inducer of radical formation. Therefore, iron supplementation may ameliorate both inflammation and oxidative stress, which are considered central factors in the dismal prognosis of dialysis patients.

Another important question is whether an upper threshold level for iron loading in dialysis patients can be identified, above which detrimental effects of iron may become apparent. Iron supplementation and a subsequent increase in ferritin levels to up to 600–800 ng/mL appeared to be safe in our study cohort. We found decreased all-cause mortality and a trend to decreased cardiovascular and sepsis-related mortality in patients with normal CRP levels. In patients with concomitantly elevated CRP we observed a trend toward a higher mortality rate in subjects with ferritin levels above 800 ng/mL. However, the significance of this observation is limited by the small number of measurements. The upper threshold for ferritin levels as an indicator of iron-mediated toxicity in dialysis patients is not yet defined. Studies comparing the effect of iron supplementation with different ferritin targets on clinical outcomes are lacking. The recently published KDIGO anemia guidelines recommend iron supplementation in patients with a transferrin saturation <30% and a serum ferritin concentration <500 ng/mL [36]. Of note, clinical data indicate a linear relationship between increased serum ferritin levels and tissue iron deposition [37]–[39]. Nevertheless, the clinical sequelae of these findings are not clear at present specifically in patients with inflammation.

Ferritin levels in the setting of inflammation are difficult to interpret, because ferritin is not only an iron storage protein but also an acute phase reactant [5], [40]–[41]. In an attempt to differentiate between these functions of time-dependent ferritin we stratified our analyses according to time-dependent normal or increased CRP levels during follow-up. We found a decreased mortality with higher ferritin concentrations in patients with normal CRP although this finding might be limited by a small sample size in this stratum of patients without inflammation. In contrast, in patients with elevated CRP as a surrogate marker of inflammation very high ferritin levels (>800 ng/ml) were associated with a slightly increased mortality risk. This relationship between high serum ferritin levels and mortality could instead be interpreted as a likely indication of more advanced inflammation and not as a consequence of iron loading because inflammation is associated with a poor outcome per se [42]. However, administration of iron under these conditions may increase radical formation as immune activation induces oxygen radical production, which is augmented by the presence of iron [14], [17], [32], [43].

A recent meta-analysis [18] as well as *in vitro* data and results from different animal models demonstrated an association between excess iron and a higher risk of infection, increased microbial virulence and growth [44]–[45] along with an impaired host immune response [15], [30], [34], [46]–[49]. However, clinical data from prospective studies are rare and produced conflicting results on the association between iron and risk for infection [16], [26], [50]–[56]. However, in our study we found a lower risk for cardiovascular or sepsis-related mortality with borderline significance in iron-treated patients.

### Strengths and limitations

The prospective recruitment of all patients starting dialysis treatment over a period of six years in a clearly circumscribed region allowed evaluation and follow-up over a long observation period without significant loss of data. Therefore, the most important bias of cross-sectional studies with a mix of prevalent and incident cases and the resulting survival bias can be excluded. A limitation of this study is the relatively small number of patients and events, which limit the number of variables for which the analysis can be adjusted. However, there were no differences between the age- and sex-adjusted models and the models with extended adjustment. Even if our sample size of more than 200 patients is small, our study might be a stimulus for larger studies employing data with this depth (duration of observation and granularity of data points).

Although biologically sound, this observation is limited by the comparably small number of patients not receiving iron supplementation. Nowadays however, almost all dialysis patients receive some sort of iron supplementation and studies with a group of patients not receiving iron therapy for years are unlikely to be conducted in the future. We also had a smaller number of diabetic patients in the group without iron supplementation. A diet low in iron, reduced gastrointestinal absorption and increased gastrointestinal blood loss may result in a higher rate of iron deficiency in patients with diabetes mellitus [57]. Assuming that patients not needing iron supplementation were probably healthier than their counterparts, our results are even more surprising. Finally, we have no definite information, why patients were selected not to receive iron supplementation. Comparable CRP values during the follow-up between patients with and without iron supplementation (median 0.74 *vs* 0.60 mg/dL, p = 0.4) argue against a higher prevalence of inflammatory or infectious diseases in patients not receiving iron supplementation. However, some kind of selection bias cannot be excluded with certainty.

### Conclusion

Iron supplementation is associated with a decreased all-cause mortality risk in incident dialysis patients. Our findings provide cautious support for the safety and benefit of judicious iron supplementation to achieve serum ferritin levels of up to approximately 600–800 ng/mL. Additional studies are needed to determine the influence of various iron dosing regimens and ferritin threshold targets on clinical outcomes such as cardiovascular disease, infection and mortality.

## Supporting Information

Figure S1 **Cox regression results.** P-splines to explore the functional form of the effect of ferritin values (ng/mL) on the log hazard ratio for the risk of all-cause mortality (a, b) and cardiovascular or sepsis-related mortality (c, d) in patients with C-reactive protein <1 mg/dL and ≧1 mg/dL during follow-up, adjusted for age, sex, diabetes mellitus and time-dependent albumin and hemoglobin. Dashed lines are the pointwise 95% CI. The rugplot at the bottom of the figures displays the number of measurements.
(DOCX)

Figure S2 **Cox regression results.** P-splines to explore the functional form of the effect of ferritin values (ng/mL) on the log hazard ratio for the risk of all-cause mortality (a, b) and cardiovascular or sepsis-related mortality (c, d) in patients with C-reactive protein <5 mg/dL and ≧5 mg/dL during follow-up, adjusted for age, sex, diabetes mellitus and time-dependent albumin and hemoglobin. Dashed lines are the pointwise 95% CI. The rugplot at the bottom of the figures displays the number of measurements.
(DOCX)

Table S1 **Association between time-dependent ferritin and all-cause mortality and cardiovascular or sepsis-related mortality in patients with C-reactive protein <1 mg/dL and ≧1 mg/dL during follow-up using time-dependent Cox proportional hazards models.**
(DOCX)

Table S2 **Association between time-dependent ferritin and all-cause mortality and cardiovascular or sepsis-related mortality in patients with C-reactive protein <5 mg/dL and ≧5 mg/dL during follow-up using time-dependent Cox proportional hazards models.**
(DOCX)

## References

[1] Koch KM, Bechstein PB, Fassbinder W, Kaltwasser P, Schoeppe W, et al (1976) Occult blood loss and iron balance in chronic renal failure. Proc Eur Dial Transplant Assoc 12:362–369. 1084526

[2] Lindsay RM, Burton JA, King P, Davidson JF, Boddy K, et al (1973) The measurement of dialyzer blood loss. Clin Nephrol 1:24–28. 4767351

[3] Goch J, Birgegard G, Danielson BG, Wikstrom B (1996) Iron absorption in patients with chronic uremia on maintenance hemodialysis and in healthy volunteers measured with a simple oral iron load test. Nephron 73:403–406. 8832597 10.1159/000189100

[4] Eschbach JW, Adamson JW (1999) Iron overload in renal failure patients: changes since the introduction of erythropoietin therapy. Kidney Int Suppl 69: S35–43. 10.1046/j.1523-1755.1999.055suppl.69035.x 10084284

[5] Weiss G, Goodnough LT (2005) Anemia of chronic disease. N Engl J Med 352:1011–1023. 15758012 10.1056/NEJMra041809

[6] Carrera F, Lok CE, de Francisco A, Locatelli F, Mann JF, et al (2010) Maintenance treatment of renal anaemia in haemodialysis patients with methoxy polyethylene glycol-epoetin beta versus darbepoetin alfa administered monthly: a randomized comparative trial. Nephrol Dial Transplant 25:4009–4017. 20522670 10.1093/ndt/gfq305 PMC2989790

[7] Deicher R, Horl WH (2003) Anaemia as a risk factor for the progression of chronic kidney disease. Curr Opin Nephrol Hypertens 12:139–143. 12589173 10.1097/00041552-200303000-00003

[8] Macdougall IC (2008) Novel erythropoiesis-stimulating agents: a new era in anemia management. Clin J Am Soc Nephrol 3:200–207. 18077782 10.2215/CJN.03840907

[9] Goodnough LT, Nemeth E, Ganz T (2010) Detection, evaluation, and management of iron-restricted erythropoiesis. Blood 116:4754–4761. 20826717 10.1182/blood-2010-05-286260

[10] Horl WH (2013) Anaemia management and mortality risk in chronic kidney disease. Nat Rev Nephrol 9:291–301. 23438972 10.1038/nrneph.2013.21

[11] Feldman HI, Santanna J, Guo W, Furst H, Franklin E, et al (2002) Iron administration and clinical outcomes in hemodialysis patients. J Am Soc Nephrol 13:734–744. 11856779 10.1681/ASN.V133734

[12] Kalantar-Zadeh K, Regidor DL, McAllister CJ, Michael B, Warnock DG (2005) Time-dependent associations between iron and mortality in hemodialysis patients. J Am Soc Nephrol 16:3070–3080. 16033854 10.1681/ASN.2005040423

[13] Feldman HI, Joffe M, Robinson B, Knauss J, Cizman B, et al (2004) Administration of parenteral iron and mortality among hemodialysis patients. J Am Soc Nephrol 15:1623–1632. 15153574 10.1097/01.asn.0000128009.69594.be

[14] Horl WH (2007) Clinical aspects of iron use in the anemia of kidney disease. J Am Soc Nephrol 18:382–393. 17229908 10.1681/ASN.2006080856

[15] Nairz M, Schroll A, Sonnweber T, Weiss G (2010) The struggle for iron - a metal at the host-pathogen interface. Cell Microbiol 12:1691–1702. 20964797 10.1111/j.1462-5822.2010.01529.x

[16] Teehan GS, Bahdouch D, Ruthazer R, Balakrishnan VS, Snydman DR, et al (2004) Iron storage indices: novel predictors of bacteremia in hemodialysis patients initiating intravenous iron therapy. Clin Infect Dis 38:1090–1094. 15095212 10.1086/382878

[17] Vaziri ND (2013) Understanding iron: promoting its safe use in patients with chronic kidney failure treated by hemodialysis. Am J Kidney Dis 61:992–1000. 23375852 10.1053/j.ajkd.2012.10.027

[18] Litton E, Xiao J, Ho KM (2013) Safety and efficacy of intravenous iron therapy in reducing requirement for allogeneic blood transfusion: systematic review and meta-analysis of randomised clinical trials. BMJ 347:f4822. 23950195 10.1136/bmj.f4822 PMC3805480

[19] Sturm G, Lamina C, Zitt E, Lhotta K, Lins F, et al (2010) Sex-specific association of time-varying haemoglobin values with mortality in incident dialysis patients. Nephrol Dial Transplant 25:2715–2722. 20190241 10.1093/ndt/gfq101

[20] Zitt E, Lamina C, Sturm G, Knoll F, Lins F, et al (2011) Interaction of time-varying albumin and phosphorus on mortality in incident dialysis patients. Clin J Am Soc Nephrol 6:2650–2656. 21903986 10.2215/CJN.03780411 PMC3359564

[21] Bailie GR, Larkina M, Goodkin DA, Li Y, Pisoni RL, et al (2014) Data from the Dialysis Outcomes and Practice Patterns Study validate an association between high intravenous iron doses and mortality. Kidney Int doi: 10.1038/ki.2014.275. [Epub ahead of print] 10.1038/ki.2014.275 25075769

[22] Horl WH (2005) Anemia and its treatment in peritoneal dialysis patients. Wien Klin Wochenschr 117 Suppl 6: 69–72. 16437336 10.1007/s00508-005-0486-9

[23] Bailie GR, Larkina M, Goodkin DA, Li Y, Pisoni RL, et al (2013) Variation in intravenous iron use internationally and over time: the Dialysis Outcomes and Practice Patterns Study (DOPPS). Nephrol Dial Transplant 28:2570–2579. 24078642 10.1093/ndt/gft062

[24] Koulouridis I, Alfayez M, Trikalinos TA, Balk EM, Jaber BL (2013) Dose of erythropoiesis-stimulating agents and adverse outcomes in CKD: a metaregression analysis. Am J Kidney Dis 61:44–56. 22921639 10.1053/j.ajkd.2012.07.014 PMC3525813

[25] Szczech LA, Barnhart HX, Inrig JK, Reddan DN, Sapp S, et al (2008) Secondary analysis of the CHOIR trial epoetin-alpha dose and achieved hemoglobin outcomes. Kidney Int 74:791–798. 18596733 10.1038/ki.2008.295 PMC2902279

[26] Brookhart MA, Freburger JK, Ellis AR, Wang L, Winkelmayer WC, et al (2013) Infection Risk with Bolus versus Maintenance Iron Supplementation in Hemodialysis Patients. J Am Soc Nephrol 24:1151–1158. 23787911 10.1681/ASN.2012121164 PMC3699831

[27] Kshirsagar AV, Freburger JK, Ellis AR, Wang L, Winkelmayer WC, et al (2013) Intravenous iron supplementation practices and short-term risk of cardiovascular events in hemodialysis patients. PLoS One 8:e78930. 24223866 10.1371/journal.pone.0078930 PMC3815308

[28] Macdougall IC, Geisser P (2013) Use of intravenous iron supplementation in chronic kidney disease: an update. Iran J Kidney Dis 7:9–22. 23314137

[29] Fell LH, Zawada AM, Rogacev KS, Seiler S, Fliser D, et al (2014) Distinct immunologic effects of different intravenous iron preparations on monocytes. Nephrol Dial Transplant 29:809–822. 24523357 10.1093/ndt/gft524 PMC3967833

[30] Sonnweber T, Theurl I, Seifert M, Schroll A, Eder S, et al (2011) Impact of iron treatment on immune effector function and cellular iron status of circulating monocytes in dialysis patients. Nephrol Dial Transplant 26:977–987. 20826742 10.1093/ndt/gfq483

[31] Gupta A, Zhuo J, Zha J, Reddy S, Olp J, et al (2010) Effect of different intravenous iron preparations on lymphocyte intracellular reactive oxygen species generation and subpopulation survival. BMC Nephrology 11:16–20. 20716362 10.1186/1471-2369-11-16 PMC2933673

[32] Koskenkorva-Frank TS, Weiss G, Koppenol WH, Burckhardt S (2013) The complex interplay of iron metabolism, reactive oxygen species, and reactive nitrogen species: Insights into the potential of various iron therapies to induce oxidative and nitrosative stress. Free Radic Biol Med 65:1174–1194. 24036104 10.1016/j.freeradbiomed.2013.09.001

[33] Weiss G, Kronenberg F (2014) Intravenous iron administration: new observations and time for the next steps. Kidney Int. In press.. 10.1038/ki.2014.324 25549119

[34] Oexle H, Kaser A, Most J, Bellmann-Weiler R, Werner ER, et al (2003) Pathways for the regulation of interferon-gamma-inducible genes by iron in human monocytic cells. J Leukoc Biol 74:287–294. 12885946 10.1189/jlb.0802420

[35] Weiss G, Meusburger E, Radacher G, Garimorth K, Neyer U, et al (2003) Effect of iron treatment on circulating cytokine levels in ESRD patients receiving recombinant human erythropoietin. Kidney Int 64:572–578. 12846752 10.1046/j.1523-1755.2003.00099.x

[36] Kidney Disease: Improving Global Outcomes (KDIGO) Anemia Work Group (2012) KDIGO Clinical Practice Guideline for Anemia in Chronic Kidney Disease. Kidney Int., Suppl. 2279–335.

[37] Canavese C, Bergamo D, Ciccone G, Longo F, Fop F, et al (2004) Validation of serum ferritin values by magnetic susceptometry in predicting iron overload in dialysis patients. Kidney Int 65:1091–1098. 14871430 10.1111/j.1523-1755.2004.00480.x

[38] Ferrari P, Kulkarni H, Dheda S, Betti S, Harrison C, et al (2011) Serum iron markers are inadequate for guiding iron repletion in chronic kidney disease. Clin J Am Soc Nephrol 6:77–83. 20876673 10.2215/CJN.04190510 PMC3022252

[39] Rostoker G, Griuncelli M, Loridon C, Couprie R, Benmaadi A, et al (2012) Hemodialysis-associated hemosiderosis in the era of erythropoiesis-stimulating agents: a MRI study. Am J Med 125:991–999. 22998881 10.1016/j.amjmed.2012.01.015

[40] Knovich MA, Storey JA, Coffman LG, Torti SV, Torti FM (2009) Ferritin for the clinician. Blood Rev 23:95–104. 18835072 10.1016/j.blre.2008.08.001 PMC2717717

[41] Thomas C, Thomas L (2005) Anemia of chronic disease: pathophysiology and laboratory diagnosis. Lab Hematol 11:14–23. 15790548

[42] Meuwese CL, Snaedal S, Halbesma N, Stenvinkel P, Dekker FW, et al (2011) Trimestral variations of C-reactive protein, interleukin-6 and tumour necrosis factor-alpha are similarly associated with survival in haemodialysis patients. Nephrol Dial Transplant 26:1313–1318. 20846939 10.1093/ndt/gfq557

[43] Tovbin D, Mazor D, Vorobiov M, Chaimovitz C, Meyerstein N (2002) Induction of protein oxidation by intravenous iron in hemodialysis patients: role of inflammation. Am J Kidney Dis 40:1005–1012. 12407646 10.1053/ajkd.2002.36334

[44] Brewster UC, Perazella MA (2004) Intravenous iron and the risk of infection in end-stage renal disease patients. Semin Dial 17:57–60. 14717813 10.1111/j.1525-139x.2004.17115.x

[45] Nairz M, Schleicher U, Schroll A, Sonnweber T, Theurl I, et al (2013) Nitric oxide-mediated regulation of ferroportin-1 controls macrophage iron homeostasis and immune function in Salmonella infection. J Exp Med 210:855–87343. 23630227 10.1084/jem.20121946 PMC3646493

[46] van Asbeck BS, Marx JJ, Struyvenberg A, Verhoef J (1984) Functional defects in phagocytic cells from patients with iron overload. J Infect 8:232–240. 6736664 10.1016/s0163-4453(84)93955-0

[47] Sengoelge G, Kletzmayr J, Ferrara I, Perschl A, Horl WH, et al (2003) Impairment of transendothelial leukocyte migration by iron complexes. J Am Soc Nephrol 14:2639–2644. 14514743 10.1097/01.asn.0000087087.61306.4a

[48] Patruta SI, Edlinger R, Sunder-Plassmann G, Horl WH (1998) Neutrophil impairment associated with iron therapy in hemodialysis patients with functional iron deficiency. J Am Soc Nephrol 9:655–663. 9555668 10.1681/ASN.V94655

[49] Deicher R, Ziai F, Cohen G, Mullner M, Horl WH (2003) High-dose parenteral iron sucrose depresses neutrophil intracellular killing capacity. Kidney Int 64:728–736. 12846772 10.1046/j.1523-1755.2003.00125.x

[50] Besarab A, Amin N, Ahsan M, Vogel SE, Zazuwa G, et al (2000) Optimization of epoetin therapy with intravenous iron therapy in hemodialysis patients. J Am Soc Nephrol 11:530–538. 10703677 10.1681/ASN.V113530

[51] Kessler M, Hoen B, Mayeux D, Hestin D, Fontenaille C (1993) Bacteremia in patients on chronic hemodialysis. A multicenter prospective survey. Nephron 64:95–100. 8502343 10.1159/000187285

[52] Hoen B, Kessler M, Hestin D, Mayeux D (1995) Risk factors for bacterial infections in chronic haemodialysis adult patients: a multicentre prospective survey. Nephrol Dial Transplant 10:377–381. 7792034

[53] Seifert A, von Herrath D, Schaefer K (1987) Iron overload, but not treatment with desferrioxamine favours the development of septicemia in patients on maintenance hemodialysis. Q J Med 65:1015–1024. 3455553

[54] Tielemans CL, Lenclud CM, Wens R, Collart FE, Dratwa M (1989) Critical role of iron overload in the increased susceptibility of haemodialysis patients to bacterial infections. Beneficial effects of desferrioxamine. Nephrol Dial Transplant 4:883–887. 2515493 10.1093/ndt/4.10.883

[55] Boelaert JR, Daneels RF, Schurgers ML, Matthys EG, Gordts BZ, et al (1990) Iron overload in haemodialysis patients increases the risk of bacteraemia: a prospective study. Nephrol Dial Transplant 5:130–134. 2113211 10.1093/ndt/5.2.130

[56] Hoen B, Paul-Dauphin A, Hestin D, Kessler M (1998) EPIBACDIAL: a multicenter prospective study of risk factors for bacteremia in chronic hemodialysis patients. J Am Soc Nephrol 9:869–876. 9596085 10.1681/ASN.V95869

[57] Stevens PE (2012) Anaemia, diabetes and chronic kidney disease: where are we now? J Ren Care 38 Suppl 1: 67–77. 22348366 10.1111/j.1755-6686.2012.00281.x

