# Supplementary figures and images for: Iron Supplementation and Mortality in Incident Dialysis Patients: An Observational Study
Source: PLoS One. 2014 Dec 2;9(12):e114144. doi: 10.1371/journal.pone.0114144 (PMC4252084; doi:10.1371/journal.pone.0114144)

**Figure S1.**


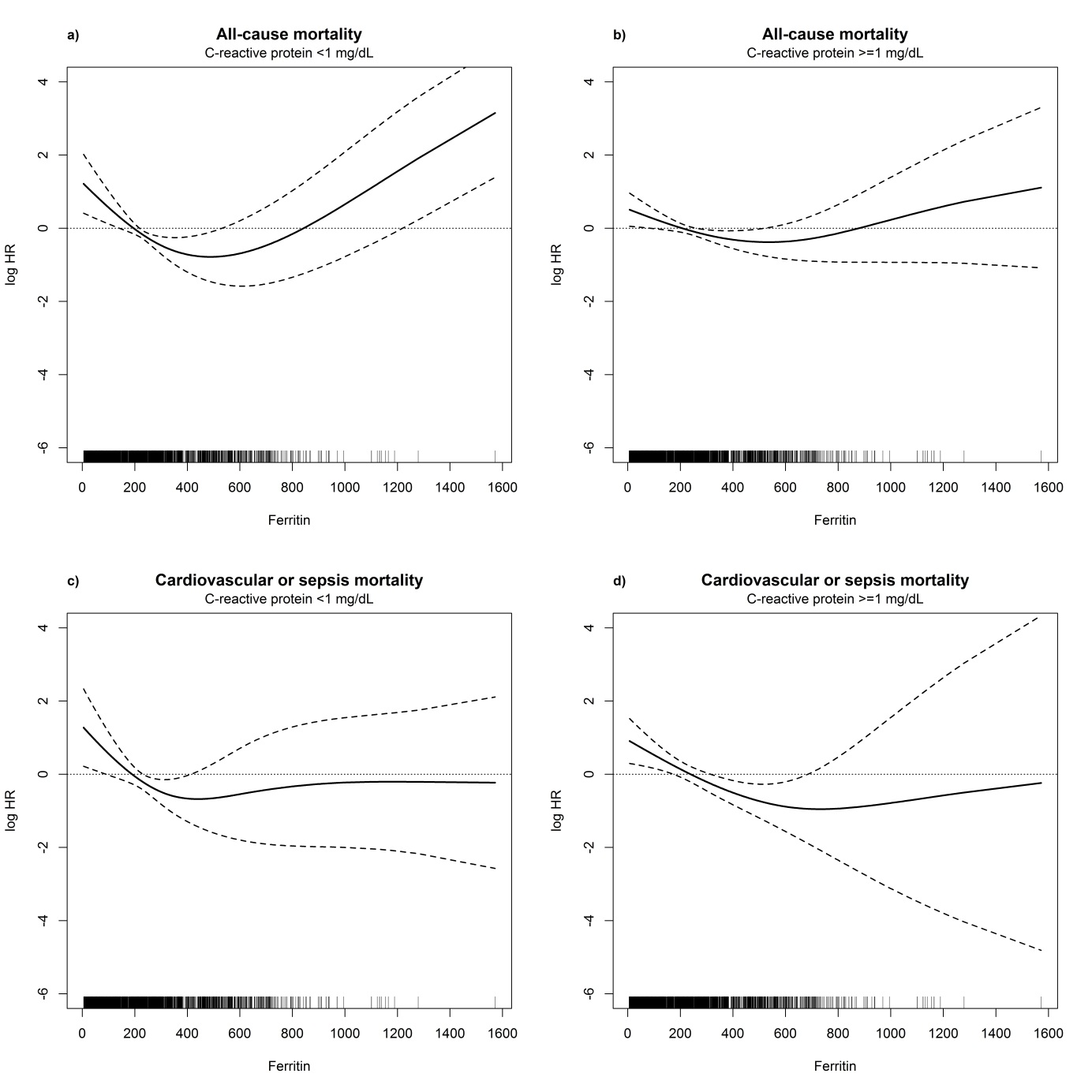

Supplement: Figure S1 — Cox regression results. P-splines to explore the functional form of the effect of ferritin values (ng/mL) on the log hazard ratio for the risk of all-cause mortality (a, b) and cardiovascular or sepsis-related mortality (c, d) in patients with C-reactive protein <1 mg/dL and ≧1 mg/dL during follow-up, adjusted for age, sex, diabetes mellitus and time-dependent albumin and hemoglobin. Dashed lines are the pointwise 95% CI. The rugplot at the bottom of the figures displays the number of measurements. (DOCX) [file pone.0114144.s001.docx]

**
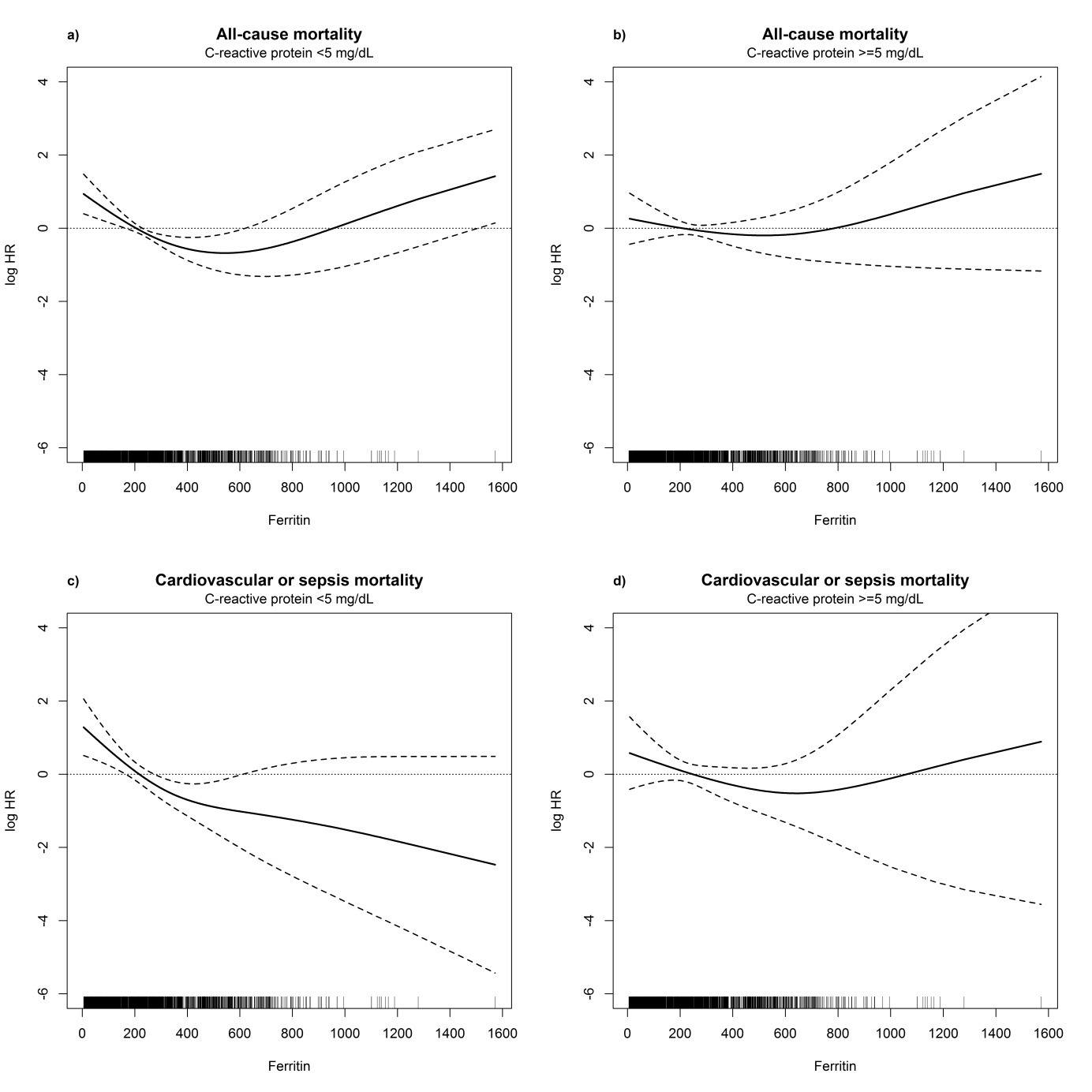
Figure S2.**

Supplement: Figure S2 — Cox regression results. P-splines to explore the functional form of the effect of ferritin values (ng/mL) on the log hazard ratio for the risk of all-cause mortality (a, b) and cardiovascular or sepsis-related mortality (c, d) in patients with C-reactive protein <5 mg/dL and ≧5 mg/dL during follow-up, adjusted for age, sex, diabetes mellitus and time-dependent albumin and hemoglobin. Dashed lines are the pointwise 95% CI. The rugplot at the bottom of the figures displays the number of measurements. (DOCX) [file pone.0114144.s002.docx]
