# Supplementary material for: Iron Supplementation and Mortality in Incident Dialysis Patients: An Observational Study
Source: PLoS One. 2014 Dec 2;9(12):e114144. doi: 10.1371/journal.pone.0114144 (PMC4252084; doi:10.1371/journal.pone.0114144)
Supplement: Table S1 — Association between time-dependent ferritin and all-cause mortality and cardiovascular or sepsis-related mortality in patients with C-reactive protein <1 mg/dL and ≧1 mg/dL during follow-up using time-dependent Cox proportional hazards models. (DOCX) [file pone.0114144.s003.docx]

**Table S1. Association between time-dependent ferritin and all-cause mortality and cardiovascular or sepsis-related mortality in patients with C-reactive protein <1 mg/dL and ≥1 mg/dL during follow-up using time-dependent Cox proportional hazards models.**

|  | | | **CRP <1 mg/dL** | | | | | | **CRP ≥1 mg/dL** | | | | | |
| --- | --- | --- | --- | --- | --- | --- | --- | --- | --- | --- | --- | --- | --- | --- |
| **Ferritin per 100 ng/mL increase** | | | **All-cause mortality** | | | **CV or sepsis mortality**** | | | **All-cause mortality** | | | **CV or sepsis mortality**** | | |
|  | | | (n events = 21) | | | (n events = 15) | | | (n events = 59) | | | (n events = 43) | | |
|  | | | HR | (95% CI) | P-value | HR | (95% CI) | P-value | HR | (95% CI) | P-value | HR | (95% CI) | P-value |
| **Non-linear effect modeling using P-splines** | | |  |  |  |  |  |  |  |  |  |  |  |  |
| Adjustment: | None | Linear part | 0.98 | (0.86-1.12) | 0.800 | 0.89 | (0.78-1.01) | 0.077 | 1.14 | (1.06-1.24) | 0.001 | 1.07 | (0.99-1.16) | 0.100 |
|  |  | Non-linear part |  |  | <0.001 |  |  | 0.010 |  |  | 0.002 |  |  | <0.001 |
|  | Age, sex | Linear part | 1.00 | (0.88-1.13) | 0.950 | 0.87 | (0.74-1.01) | 0.069 | 1.12 | (1.03-1.22) | 0.007 | 1.05 | (0.96-1.15) | 0.260 |
|  |  | Non-linear part |  |  | <0.001 |  |  | 0.009 |  |  | 0.004 |  |  | <0.001 |
|  | Extended***** | Linear part | 1.01 | (0.89-1.14) | 0.920 | 0.87 | (0.75-1.02) | 0.082 | 1.06 | (0.98-1.16) | 0.160 | 0.99 | (0.91-1.08) | 0.860 |
|  |  | Non-linear part |  |  | <0.001 |  |  | 0.002 |  |  | 0.001 |  |  | <0.001 |

Shown for each model are estimated HRs for the linear component of the non-linear P-spline and HRs for ferritin measurements per 100 ng/mL increase.

***** Adjusted for age, sex, diabetes mellitus and time-dependent albumin and hemoglobin.

** **Cardiovascular or sepsis mortality**: myocardial infarction (MI), heart failure, sudden death, ischemic stroke, hemorrhagic stroke, sepsis.

*Note:* No conversion necessary for ferritin in ng/mL and µg/L.
