# Supplementary material for: Iron Supplementation and Mortality in Incident Dialysis Patients: An Observational Study
Source: PLoS One. 2014 Dec 2;9(12):e114144. doi: 10.1371/journal.pone.0114144 (PMC4252084; doi:10.1371/journal.pone.0114144)
Supplement: Table S2 — Association between time-dependent ferritin and all-cause mortality and cardiovascular or sepsis-related mortality in patients with C-reactive protein <5 mg/dL and ≧5 mg/dL during follow-up using time-dependent Cox proportional hazards models. (DOCX) [file pone.0114144.s004.docx]

**Table S2. Association between time-dependent ferritin and all-cause mortality and cardiovascular or sepsis-related mortality in patients with C-reactive protein <5 mg/dL and ≥5 mg/dL during follow-up using time-dependent Cox proportional hazards models.**

|  | | | **CRP <5 mg/dL** | | | | | | **CRP ≥5 mg/dL** | | | | | |
| --- | --- | --- | --- | --- | --- | --- | --- | --- | --- | --- | --- | --- | --- | --- |
| **Ferritin per 100 ng/mL increase** | | | **All-cause mortality** | | | **CV or sepsis mortality**** | | | **All-cause mortality** | | | **CV or sepsis mortality**** | | |
|  | | | (n events = 49) | | | (n events = 35) | | | (n events = 31) | | | (n events = 23) | | |
|  | | | HR | (95% CI) | P-value | HR | (95% CI) | P-value | HR | (95% CI) | P-value | HR | (95% CI) | P-value |
| **Non-linear effect modeling using P-splines** | | |  |  |  |  |  |  |  |  |  |  |  |  |
| Adjustment: | None | Linear part | 1.10 | (1.02-1.18) | 0.017 | 0.76 | (0.71-0.83) | <0.001 | 1.24 | (1.13-1.36) | <0.001 | 1.16 | (1.07-1.27) | <0.001 |
|  |  | Non-linear part |  |  | <0.001 |  |  | 0.095 |  |  | 0.007 |  |  | <0.001 |
|  | Age, sex | Linear part | 1.10 | (1.01-1.19) | 0.025 | 0.75 | (0.69-0.82) | <0.001 | 1.20 | (1.09-1.33) | <0.001 | 1.16 | (1.05-1.27) | 0.004 |
|  |  | Non-linear part |  |  | <0.001 |  |  | 0.052 |  |  | 0.014 |  |  | <0.001 |
|  | Extended***** | Linear part | 1.05 | (0.96-1.13) | 0.290 | 0.73 | (0.66-0.80) | <0.001 | 1.15 | (1.02-1.30) | 0.023 | 1.10 | (1.00-1.22) | 0.045 |
|  |  | Non-linear part |  |  | 0.001 |  |  | 0.005 |  |  | 0.012 |  |  | <0.001 |

Shown for each model are estimated HRs for the linear component of the non-linear P-spline and HRs for ferritin measurements per 100 ng/mL increase.

***** Adjusted for age, sex, diabetes mellitus and time-dependent albumin and hemoglobin.

** **Cardiovascular or sepsis mortality**: myocardial infarction (MI), heart failure, sudden death, ischemic stroke, hemorrhagic stroke, sepsis.

*Note:* No conversion necessary for ferritin in ng/mL and µg/L.
